# FOXF1 and SHH participate in the regulation of iron signaling in pulmonary fibrosis

**DOI:** 10.1016/j.redox.2025.103893

**Published:** 2025-10-10

**Authors:** Xue Wang, Xin Liu, Yumei Fan, Ke Tan, Jiaqi Gao, Yuejiao Wang, Ziyi Zhang, Shuyue Liu, Xiaofan Wang, Baohua Wang, Pengxiu Cao

**Affiliations:** a Ministry of Education Key Laboratory of Molecular and Cellular Biology, Hebei Key Laboratory of Animal Physiology, Biochemistry and Molecular Biology, College of Life Sciences, Hebei Normal University, Hebei Research Center of the Basic Discipline of Cell Biology, Shijiazhuang, Hebei, China; b Changli Institute of Pomology, Hebei Academy of Agriculture and Forestry Sciences, Qinhuangdao, Hebei, China; c Department of Thoracic Surgery, The Second Hospital of Hebei Medical University, Hebei, China

**Keywords:** Pulmonary fibrosis, Iron, FOXF1, Redox balance, FDX1, SHH

## Abstract

Pulmonary fibrosis (PF) involves persistent activation of fibroblasts and excessive deposition of extracellular matrix, with limited therapeutic options. Pulmonary iron overload has been identified in PF and is associated with the progression of PF. However, the underlying signaling pathway remains unclear. This study demonstrated that iron accumulates in the mouse lung from day 7 post-bleomycin (BLM) instillation until harvest, coinciding with the activation of pulmonary fibroblasts and the onset of fibrogenesis. Iron supplementation promoted the G_1_/S cell cycle transition and proliferation of fibroblasts, and worsened PF, whereas iron deficiency demonstrated the opposite effects. Mechanistically, both iron and reactive oxygen species (ROS) suppress Forkhead box F1 (FOXF1) expression. *FOXF1* overexpression upregulates the expression of antioxidant proteins, including ferredoxin 1 (FDX1) and heme oxygenase-1 (HO-1). Both *FOXF1* overexpression and *FDX1* overexpression reduced cellular labile iron pool (LIP), ROS levels, and collagen synthesis in human pulmonary fibroblasts. Sonic hedgehog (SHH) signaling elevated intracellular iron, fibroblast proliferation, and its own secretion, establishing a sustained SHH/iron amplifying loop. These findings identify an iron/ROS–FOXF1 positive feedback loop and an SHH–iron self-promoting pathway that drive sustained elevated iron levels and persistent fibroblast activation and fibrogenesis, thereby deepening our understanding of the iron signaling in PF.

## Highlights

• Iron overload drives fibroblast proliferation and collagen deposition in pulmonary fibrosis.
• Iron/ROS–FOXF1 positive feedback and SHH–iron self-promotion sustain high iron in PF.
• FOXF1 mediates iron/ROS signaling during lung fibrogenesis.
• Iron contributes to the activation of fibroblasts by SHH signaling.

## 1 Introduction

Pulmonary fibrosis (PF) is a type of lung disease characterized by the chronic and progressive accumulation of a large amount of extracellular matrix, resulting in structural destruction by scarring and decreased lung function. It is a common pathological change in the advanced stages of various lung diseases or lung injuries [1]. Unfortunately, there are currently no drugs that can cure or reverse PF. Mesenchymal cells (CD44^+^/CD73^+^/CD105^+^/CD90^+^/CD45^-^/CD31^-^/EPCAM^−^) in the lung are a type of multipotent stromal cells [2]. Under physiological conditions, mesenchymal cells remain in a relatively quiescent state, characterized by low levels of cell proliferation and differentiation. When lung injury occurs, the synthesis and secretion of pro-inflammatory factors increase, and mesenchymal cells are activated, as evidenced by increased proliferation ability and accelerated cell cycle progression [3]. Afterwards, mesenchymal cells differentiate into fibroblasts that express high levels of extracellular matrix, resulting in the accumulation of extracellular matrix in the walls of bronchioles and microvessels, which leads to the development of fibrosis. Therefore, the activation, proliferation, and fibrotic differentiation of lung mesenchymal cells are the key factors that contribute to PF.

In the commonly used bleomycin (BLM)-induced PF (BLM-PF) mouse model, at the first stage (approximately 1–7 days) after tracheal administration of BLM, lung mesenchymal cells are mainly activated by inflammatory cytokines, and proliferation starts. The later stage (approximately 8–28 days) is characterized by the coexistence of excess proliferation and fibrotic differentiation into fibroblasts and myofibroblasts. Obviously, the proliferation of mesenchymal cells promotes the process of PF caused by abnormal repair after lung injury. However, the exact triggering factors and signaling pathways that lead to the proliferation of mesenchymal cells after lung injury remain unclear.

In recent years, pulmonary iron homeostasis imbalance, particularly pulmonary iron overload, has been found to be related to various lung diseases, including PF [[4], [5], [6], [7]]. In alveolar macrophages of patients with chronic obstructive pulmonary disease or idiopathic pulmonary fibrosis (IPF), the accumulation of iron or hemosiderin increases, and iron accumulation is associated with the deterioration of lung function [6,8]. Our previous studies have demonstrated that iron accumulates in BLM-PF mouse lungs, with higher ferritin levels persisting from day 1–21, reaching a peak around day 10–14 post-BLM instillation [7]. It has also been reported that BLM-PF is associated with an increase in Tfr1^+^ macrophages that display an altered phenotype, and both fibrosis and lung function decline are associated with pulmonary iron accumulation [9]. The consistency between the BLM-PF model and IPF patients suggests that this model can be used to study the characteristics of iron metabolism alterations in tissues and specific cell types at different stages of PF.

In addition, although some published studies have shown that iron promotes the progression of PF, another study demonstrates that iron does not exacerbate BLM-PF in experimental mice [10]. Furthermore, it has been reported that lung fibroblasts exhibit a greater proliferation rate when exposed to increased iron levels [7,9]. However, the signal transduction pathway by which iron accelerates the proliferation rate of pulmonary fibroblasts is still unknown. In this study, we aim to discover the characteristics of iron metabolism changes in lung tissues and key cell types in the BLM-PF model. Subsequently, the regulatory effects of iron on PF will be determined by administering iron supplements and iron chelating agents to experimental animals with BLM-PF, as well as utilizing BLM-induced *tfr1*^*+/−*^ mice. Importantly, we aim to dissect the iron-related signaling pathway in PF. This project will help us comprehensively understand the pathological mechanism of PF and identify effective intervention strategies targeting iron metabolism signaling pathways.

## 2 Materials and methods

### 2.1 Study approval

The collection of PF and non-PF tissues from excisional pulmonary lobules after lobectomy was approved by the Research Ethics Committee of the Second Hospital of Hebei Medical University (approval No. 2020-R331), and written informed consent was received from all patients included in this study.

### 2.2 Establishment of the BLM-induced PF mouse model and drug administration

All animal-related procedures in this study were approved by the Biomedical Ethics Committee of Hebei Normal University (approval Nos. 2019SC02 and 2020LLSC017) and complied with NIH guidelines for the Care and Use of Laboratory Animals.

Eight-week-old male C57BL/6J mice were purchased from Beijing HFK Bioscience (Beijing, China). After one week of adaptation, the animals were randomly divided into three groups: the control PBS group, the BLM-PF group, and the BLM-PF groups with different treatments. Animals were anesthetized with 5 % chloral hydrate at a dose of 5 ml/kg, followed by the instillation of 50 μL PBS or 1.5 U/kg BLM (B107423, Aladdin, Shanghai, China) in 50 μL PBS solution into the control or BLM-induced animal groups through the trachea, respectively. Starting from day 7 post-BLM instillation, 600 mg/kg ferric ammonium citrate (FAC) (F5879, Sigma, Darmstadt, Germany) or 50 mg/kg deferoxamine (DFO) (14595, Cayman, Ann Arbor, USA) were administered to the corresponding groups of animals once daily by oral gavage till sacrifice on day 21. PBS control and nontreated BLM-PF mice were given the same amount of solvent by oral gavage.

### 2.3 *Tfr1*^*+/−*^ mice

Since *tfr1*^*−/−*^ mice are embryonically lethal, *tfr1*^*+/−*^ mice were purchased from Sai Ye Biotechnology Co., Ltd. (Suzhou, China) and used in this study. The targeted mutation involves the deletion of exons 3 and 4 of the *Tfr1* gene. The primer sequences used for genotyping were as follows: Forward, AATAGGGTAGCTTTGGCTGTTTTG; Reverse, TCTGGAGAAGCAAGATCAAACACT. Genotyping by PCR amplification yielded two products (408 bp and 1911 bp) in heterozygous mice, while the wild-type (WT) allele produced a single 1911 bp product.

### 2.4 Hydroxyproline assay

A hydroxyproline assay was performed to quantify the amount of collagen in the mouse lungs. Briefly, mouse lungs were homogenized in 1 mL PBS, mixed with an equal volume of 12 N HCl, and hydrolyzed at 124 °C for 24 h. Samples or hydroxyproline standards (5 μL) were added to a 96-well plate containing 5 μL citrate/acetate buffer and 100 μL chloramine T, followed by incubation at room temperature for 30 min. After adding 100 μL Ehrlich's reagent, the plate was incubated at 65 °C for 30 min. Absorbance at 550 nm was measured, and hydroxyproline content was calculated from the standard curve.

### 2.5 Extraction of primary lung fibroblasts and cell culture

Mouse primary lung fibroblasts were isolated and cultured as described [7]. Human lung tissues from excisional pulmonary lobules were cut into small pieces by spring scissors, and then digested in serum-free DMEM medium with 0.1 % collagenase Ⅰ (C8140, Solarbio, Beijing, China) for 40 min in a shaker at 37 °C. After being filtered through a 100 μm strainer and subsequently centrifugation at 400×*g* for 5 min at 4 °C, the obtained cell pellet was suspended and seeded in cell culture dishes with DMEM supplemented with 10 % FBS (FSP500, ExCell Bio, Shanghai, China), 100 U/ml penicillin, 0.1 mg/ml streptomycin, and 0.25 μg/ml amphotericin B at 37 °C with 5 % CO_2_ in a cell incubator. Primary human pulmonary fibroblasts at passages 4–9 were used for the indicated treatments.

Raw264.7 cell line was kindly provided by the Stem Cell Bank, Chinese Academy of Sciences (Shanghai, China) and cultured in DMEM containing 10 % FBS (FSP500, ExCell Bio) at 37 °C with 5 % CO_2_ in a cell incubator.

### 2.6 RNA extraction and real-time PCR

A micro cell/tissue total RNA extraction kit (TR150-50, Tianmo Biotech, Beijing, China) was utilized to extract total RNA from the cells, and cDNA was synthesized with a NovoScript® Plus All-in-one 1st Strand cDNA Synthesis SuperMix kit (E047-01A, Novoprotein, Shanghai, China). In the real-time PCR reaction system, 1 × SYBR Green PCR premix was used, and 0.25 μM of the following gene primer pairs were applied: *Forkhead box F1 (FOXF1) (human)* forward, GCGGCTTCCGAAGGAAATG,

*FOXF1 (human)* reverse, CAAGTGGCCGTTCATCATGC;

*Ferredoxin 1(FDX1) (human)* forward, TTCAACCTGTCACCTCATCTTTG,

*FDX1 (human)* reverse, TGCCAGATCGAGCATGTCATT;

*Heme oxygenase 1(HO-1) (human)* forward, CCTTCTTCACCTTCCCCAACAT,

*HO-1 (human)* reverse, TCTTCTATCACCCTCTGCCTGA;

*β-actin (human)* forward, CGAGGACTTTGATTGCACATTG,

*β-actin (human)* reverse, ACTGGGCCATTCTCCTTAGA;

*18S rRNA (human)* forward, AGTTGGTGGAGCGATTTGT,

*18S rRNA (human)* reverse, CTTGTCCCTCTAAGAAGTTGGG;

*Hyaluronan synthase 2 (Has2) (mouse)* forward, TGTGAGAGGTTTCTATGTGTCCT,

*Has2 (mouse)* reverse, ACCGTACAGTCCAAATGAGAAGT;

*High mobility group AT-hook 2 (Hmga2) (mouse)* forward, GAGCCCTCTCCTAAGAGACCC,

*Hmga2 (mouse)* reverse, TTGGCCGTTTTTCTCCAATGG;

*18S rRNA (mouse)* forward, CATTAATCAAGAACGAAAGTCGG,

*18S rRNA* (mouse) reverse, TTAAGTTTCAGCTTTGCAACCAT. Gene expression levels were normalized to *β-actin* or *18S rRNA* as indicated using the 2^−ΔΔCT^ method.

### 2.7 Immunoblot analysis

Immunoblotting was performed as previously described [7]. The following primary antibodies were applied: anti-ferritin light chain (FTL, 1:1000, K002972P, Solarbio), anti-Ferritin (1:1000, ET1705-54, Huabio, Hangzhou, China), anti-FOXF1 (1:1000, A6513, ABclonal, Wuhan, China), anti-FDX1 (1:1000, A20895, ABclonal), anti–HO–1 (1:1000, WL02400, Wanleibio, Shenyang, China), anti-sonic hedgehog (SHH) (1:1000, K005418P, Solarbio), anti-ferritin heavy polypeptide 1 (Fth1, 1:1000, K108517P, Solarbio), anti-human CCND1 (1:1000, K002113P, Solarbio), anti-β-actin (1:1000, GB12001, Servicebio, Wuhan, China). ImageJ was used to quantify the immunoblot bands, and the average expression level of the target protein in the control samples on the same gel was normalized to 1.

### 2.8 Gene overexpression or silencing

Both gene overexpression (OE) and silencing of the indicated genes in human pulmonary fibroblasts were performed using LipoHigh transfection reagent (E607403, Songon Biotech, Shanghai, China) according to the manufacturer's instructions. FOXF1 (NM_001451.3), FDX1 (BC017063.1), and SHH (L38518.1) overexpressing vectors were established by inserting the whole coding region into the restriction sites of *Age*I/*Bam*HI, *Age*I/*Kpn*I, and *Age*I/*Bam*HI, respectively, of the pEGFP-C1 vector. The following two pairs of *siFOXF1* sequences were used in this study: *siFOXF1*-1: sense, CCGGAGAAGCCGCCCUAUUCCUACAUC; antisense, UGUAGGAAUAGGGCGGCUUCUCCGGCG. *siFOXF1*-2: sense, GAAAGGAGUUUGUCUUCUC; antisense, GAGAAGACAAACUCCUUUC.

### 2.9 Measurement of iron levels in lung tissue or serum in mice

Mouse lung tissues were homogenized in normal saline at a ratio of 1:9 (w/v), followed by centrifugation. The supernatants were collected, and total iron levels were quantified using a Tissue Iron Assay Kit (A039-2-1, Nanjing Jiancheng Bioengineering Institute, Nanjing, China) according to the manufacturer's instructions. Mouse serum iron levels were measured using a Serum Iron Assay Kit (A039-1-1, Nanjing Jiancheng Bioengineering Institute) according to the manufacturer's instructions.

### 2.10 Immunohistochemical staining

Paraffin-embedded lung tissue sections were deparaffinized in xylene and subjected to antigen retrieval using microwave heating in sodium citrate buffer. Endogenous peroxidase activity was blocked with 3 % hydrogen peroxide, followed by a 30-min incubation with 5 % bovine serum albumin. The following primary antibodies were applied: anti-Ftl (1:100, K002972P, Solarbio), anti-Fth1 (1:100, K108517P, Solarbio), anti-FOXF1 (1:100, A03563-1-50 μL, Boster, Wuhan, China), and anti-SHH (1:100, K005418P, Solarbio). Sections were incubated overnight at 4 °C with the corresponding primary antibody, washed with PBS, and then incubated with the appropriate secondary antibodies at room temperature for 50 min. After PBS washes, chromogenic detection was performed using DAB substrate, followed by hematoxylin counterstaining and dehydration. Slides were mounted using neutral resin. Images were acquired using an Olympus BX43 upright microscope equipped with an Olympus U-LH50HG imaging system.

### 2.11 Immunofluorescence staining

Immunofluorescence staining was conducted as previously described [7]. The primary antibodies used included anti-α-smooth muscle actin (α-SMA) (1:100, ARG66381, Arigo Biolaboratories, Shanghai, China) and anti-FOXF1 (1:100, A03563-1-50 μL, Boster).

### 2.12 Measurement of the intracellular labile iron pool (LIP) level

Primary human lung fibroblasts were cultured in 6-cm dishes and treated accordingly for 24 h. Cells were harvested by trypsinization, centrifuged at 5000 rpm for 5 min at 4 °C (Fresco 21, Thermo Scientific, Waltham, MA), washed with PBS, and resuspended in PBS. The cells were then incubated with FeRhoNox-1, a Fe^2+^-sensitive fluorescent probe, at a concentration of 5 μM, at 37 °C in the dark for 30 min. After staining, cells were filtered through a 100 μm mesh and analyzed using a CytoFLEX flow cytometer (A00-1-1102, Beckman Coulter, Brea, USA) with excitation at 488 nm and fluorescence detection at the channel of 585 nm.

### 2.13 Measurement of reactive oxygen species (ROS)

Human lung fibroblasts treated with the compounds for 24 h were trypsinized and centrifuged at 5000 rpm for 5 min at 4 °C (Fresco 21, Thermo Scientific). Cells were washed twice with PBS and resuspended in 1 mL PBS. Then, the cells were incubated at 37 °C with 2′,7′-dichlorodihydrofluorescein diacetate (DCFH-DA) at a concentration of 1 μM in the dark for 30 min. After additional washing and filtration through a 100 μm cell mesh, intracellular ROS levels were measured using a CytoFLEX flow cytometer (A00-1-1102, Beckman Coulter) with excitation at 488 nm and fluorescence detection at the channel of 525 nm.

### 2.14 Cell cycle assay

Cell cycle distribution was evaluated using a Cell Cycle Detection Kit (WLA010a, Wanlei Bio, Shenyang, China) following the manufacturer's instructions. Briefly, primary human lung fibroblasts treated with drugs for 24 h were collected by trypsin digestion, centrifuged at 3200 rpm for 5 min at 4 °C (Fresco 21, Thermo Fisher Scientific, Waltham, USA), and fixed in 70 % ethanol overnight at 4 °C. After washing with PBS, the cells were incubated with RNase A at 37 °C for 30 min, followed by staining with propidium iodide at 4 °C in the dark for an additional 30 min. Samples were filtered through a 100 μm mesh and analyzed using a CytoFLEX flow cytometer (A00-1-1102, Beckman Coulter) with excitation at 488 nm and fluorescence detection at the channel of 585 nm. Data were processed using FlowJo software (version 10.8.1).

### 2.15 Statistics

Statistical analysis was conducted with GraphPad Prism 8.4.2 software. The mean ± SEM was presented for all data. Unless otherwise indicated, *t*-test was performed for comparisons between 2 groups, and One-way ANOVA followed by a Bonferroni multiple comparisons test was used for comparisons among more than two groups. *P* < 0.05 was considered statistically significant.

## 3 Results

### 3.1 Elevated iron levels in PF lungs and in the primary cell types of BLM-induced mice with PF

**Fig. 1:**
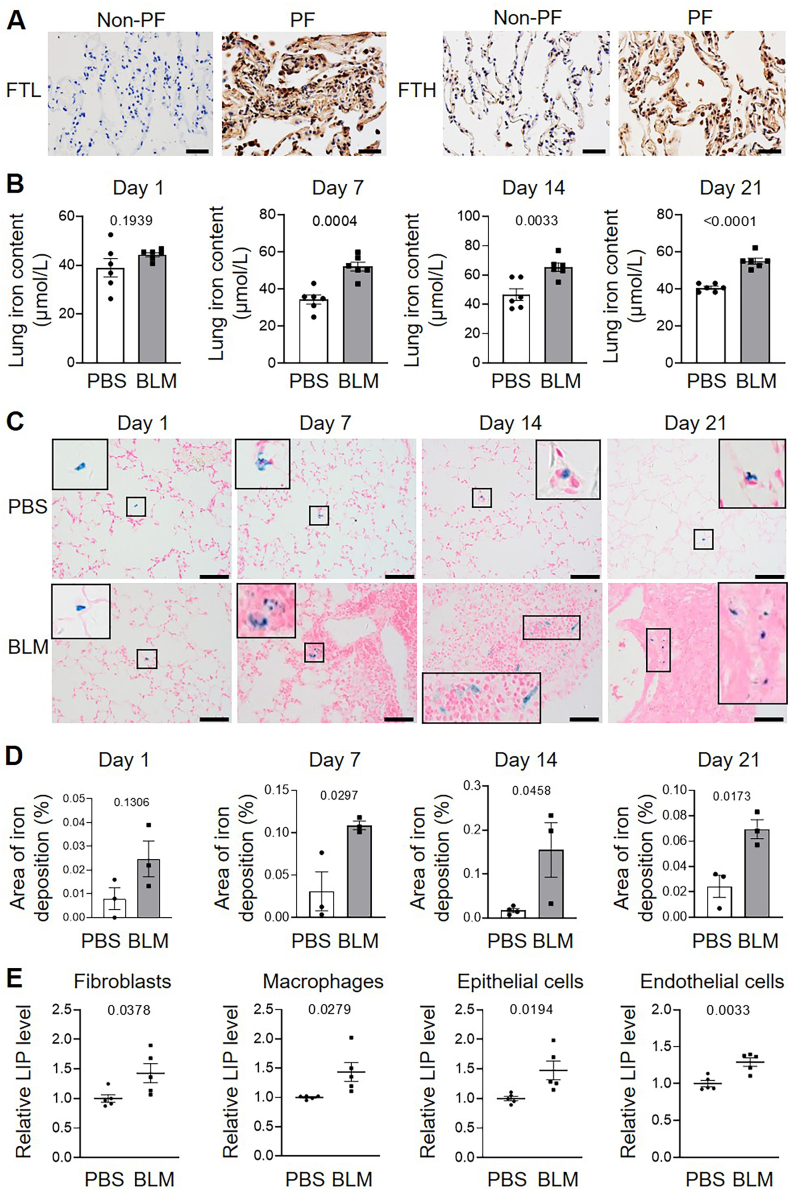
**Elevated iron levels were observed in lung tissues from PF patients and BLM-induced mice.** A, Representative immunohistochemistry images show FTL and FTH expression in non-PF and PF lung sections. n = 3/group. Scale bars, 50 μm. B, Pulmonary iron levels were measured on days 1, 7, 14, and 21 post-BLM instillation in mice. n = 6/group. C, Prussian blue staining was performed to detect hemosiderin in lung sections. The small and framed region within each image was enlarged in the larger frame. Scale bars, 50 μm. D, Quantification of the iron deposition signals in Prussian blue staining images by ImageJ. n = 3–4/group. E, Dissociated lung cells were stained with calcein acetoxymethyl ester and antibodies against fibroblasts (Cd45^−^/Pdgfrα^+^), macrophages (Cd45^+^/F4/80^+^), epithelial cells (Cd45^−^/Cd326^+^), and endothelial cells (Cd45^−^/Cd31^+^), after which calcein fluorescence was analyzed by flow cytometry to determine LIP levels. n = 5/group. Scale bars, 100 μm.

Immunohistochemical analysis revealed increased expression of FTL and ferritin heavy chain (FTH) in lung tissues from patients with PF compared to non-PF controls (Fig. 1A). Previous studies have reported that elevated iron levels alleviate the translational inhibition of FTL and FTH [7]. These findings suggest that iron accumulation is present in the lungs of patients with PF.

To further investigate this observation, we established a murine model of PF via intratracheal instillation of BLM. In this model, total pulmonary iron levels were significantly increased on days 7, 14, and 21 post-instillations compared to PBS-treated controls (Fig. 1B). Similarly, hemosiderin content in lung tissue was elevated at the same time points (Fig. 1C and D). Notably, on day 21, LIP level was significantly elevated in several key pulmonary cell types, including fibroblasts, macrophages, epithelial cells, and endothelial cells, in BLM-treated mice relative to controls (Fig. 1E). Collectively, these results demonstrate that iron accumulation is a characteristic feature of both fibrotic lung tissue and multiple pulmonary cell populations in the BLM-induced mouse model of PF.

### 3.2 Iron promotes BLM-induced PF in the mouse

**Fig. 2:**
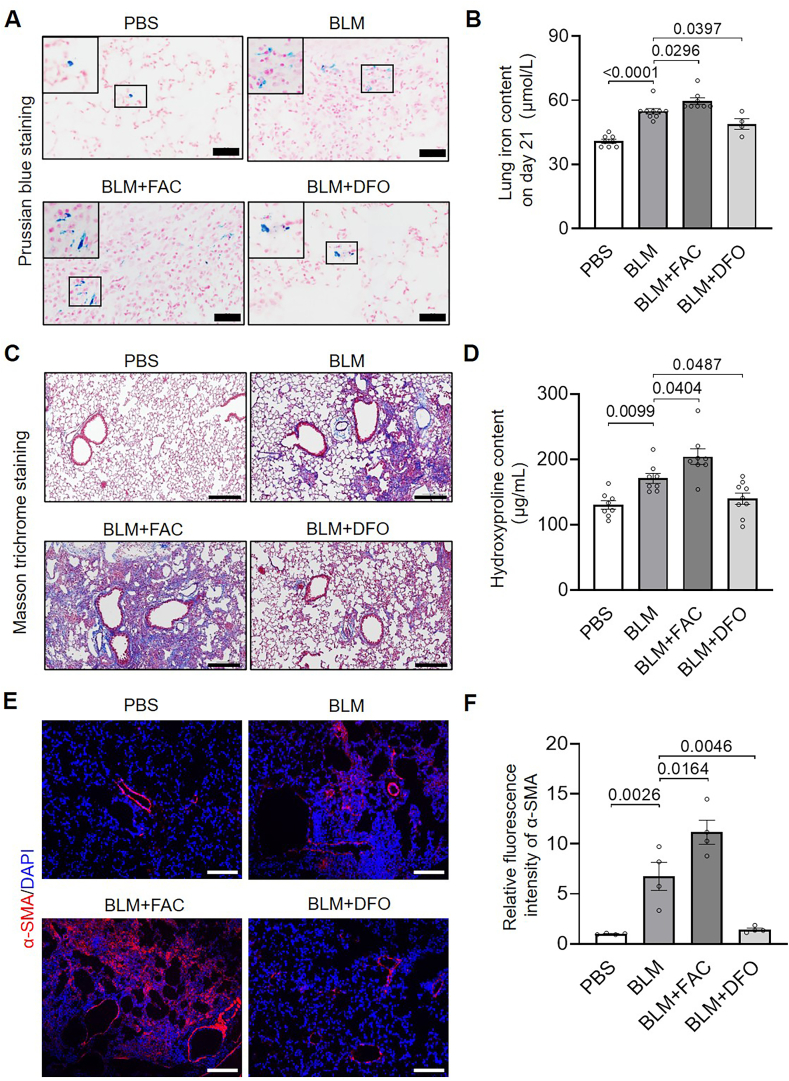
**High iron increases pulmonary iron levels and exacerbates fibrosis in BLM-PF mice, whereas iron depletion reduces iron levels and attenuates fibrosis.** From day 7 after BLM administration, mice were treated daily by oral gavage with either FAC (BLM + FAC group, 600 mg/kg) or DFO (BLM + DFO group, 50 mg/kg), and lungs were collected on day 21. A, Representative Prussian blue staining images of lung sections. For each image, the small and framed region was enlarged and shown in the larger frame. n = 5–10/group. Scale bars, 40 μm. B, Pulmonary iron levels. n = 4–9/group. C, Representative Masson's trichrome staining images. n = 5–10/group. Scale bars, 200 μm. D, Hydroxyproline content. n = 8–9/group. E and F, Representative α-SMA immunofluorescence staining images (E) and quantitative fluorescence analysis using ImageJ (F). n = 4/group. Scale bars, 100 μm.

To confirm iron plays a role in regulating the progression of PF, mice were given iron supplement FAC or iron chelator DFO by oral gavage from day 7 post-BLM instillation till harvest on day 21. It was found that FAC and DFO significantly increased and decreased the levels of hemosiderin and iron in the fibrotic mouse lungs, respectively (Fig. 2A and B). Results from Masson-trichrome staining and hydroxyproline assay demonstrated that FAC and DFO aggravated and alleviated the degree of PF and collagen content, respectively (Fig. 2C and D). Consistently, the expression of α-SMA was enhanced by FAC treatment and was reduced by DFO treatment in BLM-PF lungs (Fig. 2E and F).

**Fig. 3:**
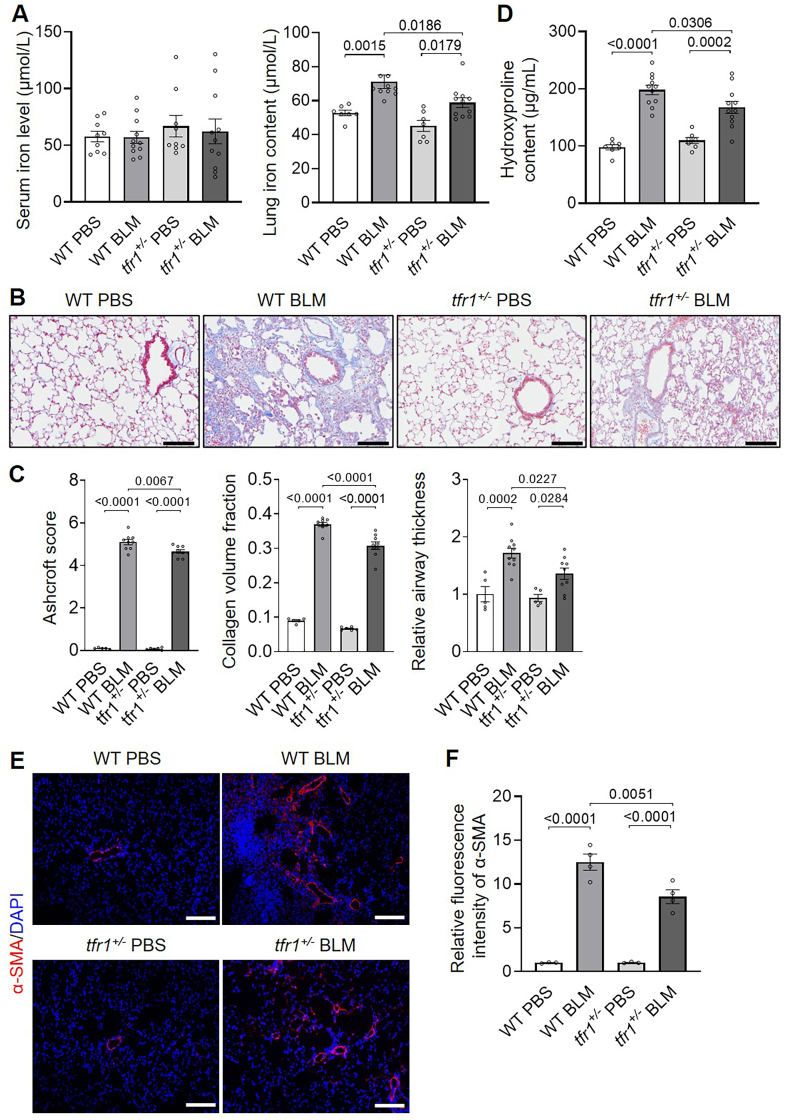
**Reduced BLM-induced lung fibrosis was observed in *tfr1*^*+/−*^ mice compared to wild-type controls**. Pulmonary fibrosis was induced in WT and *tfr1*^*+/−*^ mice by intratracheal BLM instillation, and lungs were harvested on day 21. A, Serum iron (left) and pulmonary iron (right) levels. n = 7–11/group. B, Representative Masson trichrome staining images. n = 5–10/group. Scale bars, 100 μm. C, Fibrosis scoring using the Ashcroft scale, along with quantification of collagen volume and airway wall thickness by VVsub using ImageJ. n = 5–10/group. D, Hydroxyproline content assay. n = 7–11/group. E and F, Representative α-SMA immunofluorescence images (E) and quantitative analysis (F). n = 3–4/group. Scale bars, 100 μm.

Moreover, *tfr1*^*+/−*^ mice were utilized further to confirm the regulatory role of iron on BLM-PF. No significant difference in either basal or BLM-induced serum iron levels was observed between WT and *tfr1*^*+/−*^ mice (Fig. 3A); while *tfr1*^*+/−*^ mice with BLM instillation have significantly lower pulmonary iron levels than that of wild-type mice (Fig. 3A), and demonstrated attenuated PF by pulmonary structure and subsequent PF parameter analysis, including Ashcroft score, collagen volume fraction and relative airway thickness by VVsub (Fig. 3B and C). Furthermore, collagen content measured by hydroxyproline assay (Fig. 3D) and α-SMA level demonstrated by fluorescent staining were also significantly reduced (Fig. 3E and F) in BLM-induced *tfr1*^*+/−*^ mice compared to that of the WT mice. Therefore, these results demonstrated that pulmonary iron promotes PF, and lowering abnormally high lung iron levels protects against PF.

### 3.3 Iron promotes cell proliferation and accelerates the cell cycle in lung fibroblasts

**Fig. 4:**
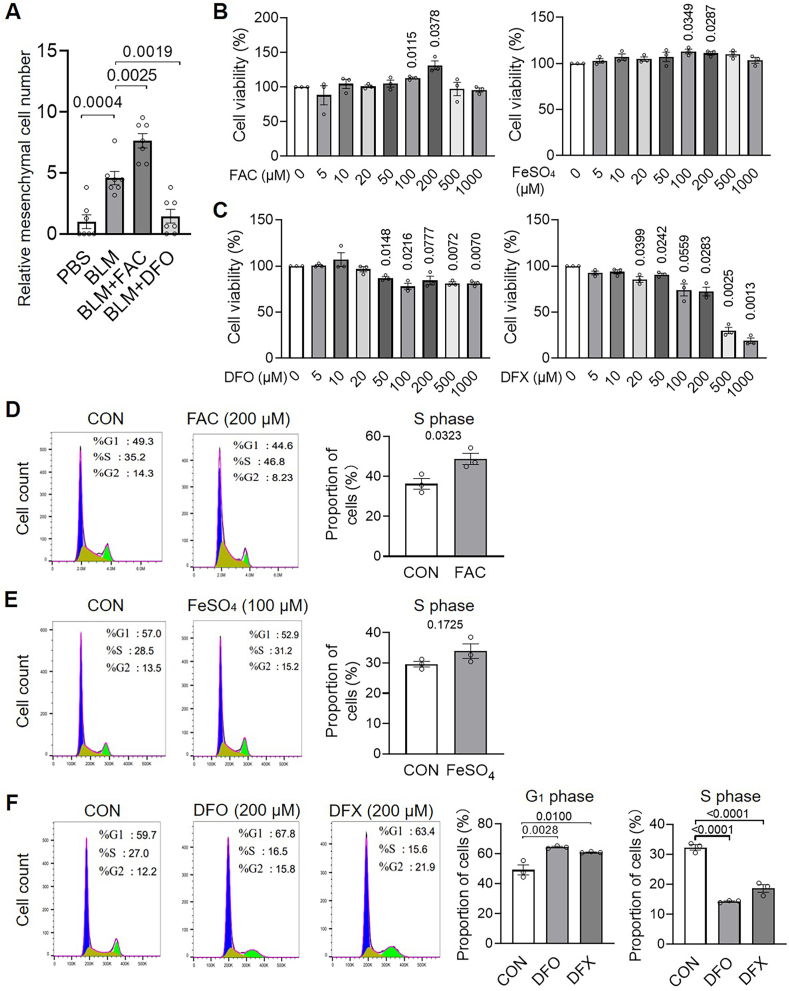
**High iron promotes cell viability and cell cycle progression, whereas iron depletion suppresses both in human primary lung fibroblasts**. A, Starting on day 7 post-BLM instillation, mice were treated with FAC (40 mg/kg) intraperitoneally or DFO (70 mg/kg) orally, until harvest on day 16. Lung sections were then immunostained with anti-Pdgfrα, and mesenchymal cells (Pdgfrα^+^) were counted from three randomly selected images at 400 × magnification per mouse. Cell numbers were normalized to the mean of the control group. n = 7/group. B and C, Human primary lung fibroblasts were treated with iron supplements (B) or iron chelators (C) for 24 h, and cell viability was determined using an MTT (3-(4,5-dimethylthiazol-2-yl)-2,5-diphenyltetrazolium bromide) Cell Proliferation and Cytotoxicity Assay kit (M1020, Solarbio) according to the manufacturer's instructions. n = 3/group. T-tests were applied for statistical analysis. D–F, Cell cycle distribution was analyzed by flow cytometry after 24 h of treatment with FAC (D), FeSO_4_ (E), or DFO/DFX (F) in human primary lung fibroblasts, and quantitative results are shown. n = 3/group.

It is interesting to understand the mechanism by which iron regulates PF. We found that FAC and DFO treatment increased and decreased the number of lung mesenchymal cells in BLM-PF mice, respectively (Fig. 4A). In vitro, 200 μM FAC and 100 μM FeSO4 enhanced, while DFO and deferasirox (DFX) inhibited the viability of primary human lung fibroblasts, respectively (Fig. 4B and C). Similarly, iron supplementation and iron chelation, respectively, enhanced and suppressed the viability of mouse lung fibroblasts in vitro (Fig. S1A and B). Furthermore, the efficacy of iron supplements and chelators was evident by higher and lower cellular LIP levels, as well as increased and decreased Ferritin expression, respectively (Fig. S2A–D). Moreover, 200 μM FAC and 100 μM FeSO4, respectively, were demonstrated to increase the cell percentages in S stages significantly or with a trend (Fig. 4D and E); Two iron chelators, DFO and DFX, both elevated the cell proportion in G_1_ stage, and reduced the cell percentages in S phase significantly (Fig. 4F). The results indicate that iron facilitates the G_1_/S checkpoint in the cell cycle of human primary lung fibroblasts, thereby promoting cell proliferation.

### 3.4 Iron inhibits Foxf1 expression both in fibrotic lungs and in vitro

**Fig. 5:**
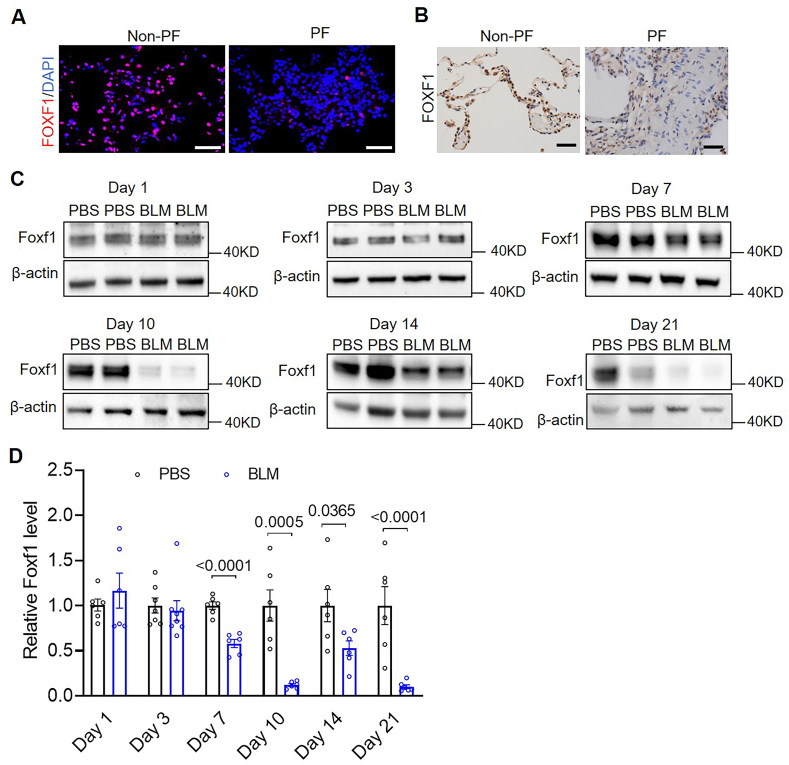
**FOXF1 expression is reduced in fibrotic lungs**. A and B, Representative immunofluorescence images of FOXF1 (A) and immunohistochemistry images (B) from non-PF and PF lung sections. Scale bars, 50 μm. C and D, Mouse lungs were collected at days 1, 3, 7, 10, 14, and 21 post-BLM instillation, and FOXF1 protein levels were analyzed by Western blot (C) and quantified using ImageJ (D). n = 6–8/group. Statistical analyses were performed using t-tests.

Then we investigated the signaling pathways through which iron regulates pulmonary fibroblast function in BLM-PF. Previous studies have documented significantly reduced FOXF1 expression in lung tissues from patients with PF, as well as in fibrotic lung-resident mesenchymal stem cells derived from the bronchoalveolar lavage fluid of transplant recipients [11]. FOXF1 is known to negatively regulate cell proliferation, cell cycle progression, and collagen synthesis [[11], [12], [13]]. We hypothesized that reduced FOXF1 expression mediates the pro-fibrotic effects of iron on lung fibroblasts. To test this, we first assessed whether FOXF1 expression is modulated by iron. Immunofluorescence and immunohistochemical analyses confirmed that FOXF1 expression was decreased in lung tissues from PF patients compared to non-PF controls (Fig. 5A and B). In the BLM-induced lung fibrosis model in mice, FOXF1 expression in lung tissue began to decline at day 7. It remained significantly suppressed through day 21 relative to PBS-treated controls (Fig. 5C and D), mirroring the temporal pattern of iron accumulation. Furthermore, FAC supplementation significantly reduced FOXF1 expression in the lungs of BLM-PF mice (Fig. S3A). Conversely, in BLM-treated *tfr1*^+/−^ mutant mice, FOXF1 expression was significantly higher than in wild-type counterparts (Fig. S3B), suggesting an inverse correlation between iron levels and FOXF1 expression in vivo.

**Fig. 6:**
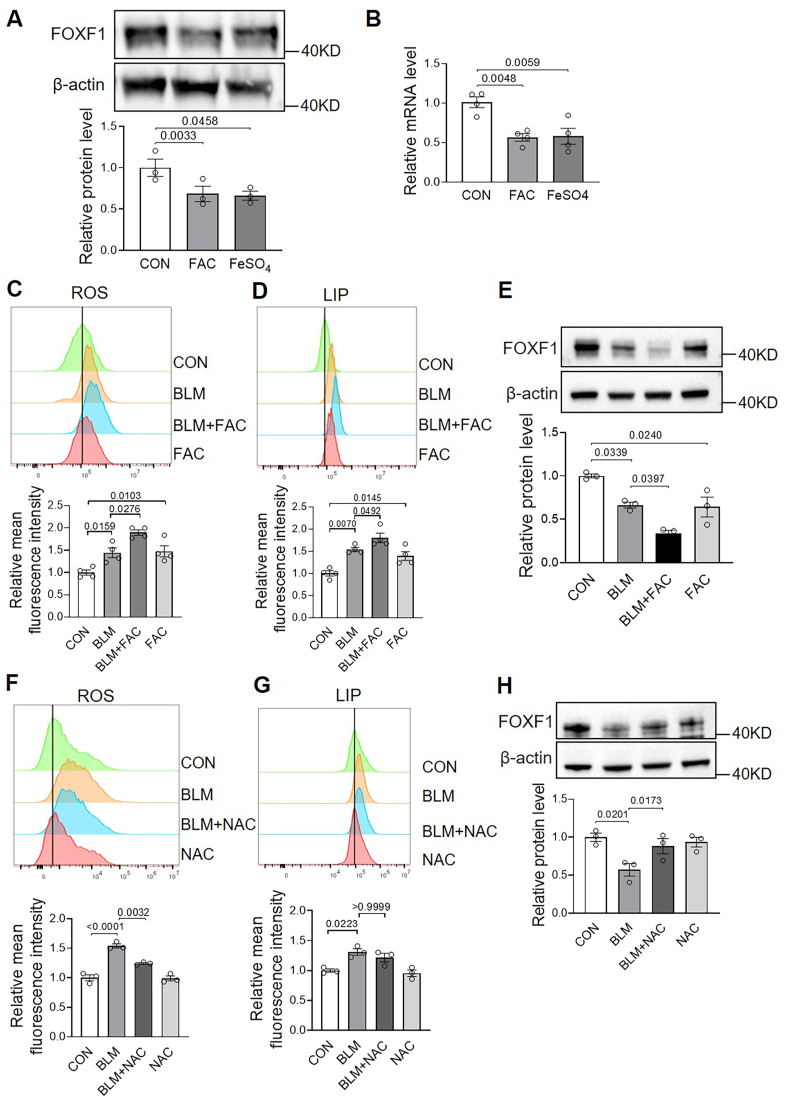
I**ron and ROS synergistically suppress FOXF1 levels in human lung fibroblasts**. Cells were treated under the indicated conditions for 24 h. A, FOXF1 protein expression and quantification following FAC or FeSO_4_ treatment. B, *FOXF1* mRNA levels determined by RT-PCR using *18S rRNA* as an internal control. C, Intracellular ROS levels measured by DCFH-DA following BLM + FAC treatment. D, LIP levels measured using FeRhoNox-1. E, FOXF1 protein expression and quantification with the indicated treatments. F, ROS levels analyzed by flow cytometry with DCFH-DA following BLM + NAC treatment. G, LIP levels determined using FeRhoNox-1 by flow cytometry. H, FOXF1 protein expression and quantification with indicated treatments. FAC (200 μM), FeSO_4_ (100 μM), BLM (40 μg/mL), NAC (2 mM); n ≥ 3/group.

Consistently, in vitro treatment of human primary lung fibroblasts with iron alone results in a significant decrease in both FOXF1 protein and mRNA levels (Fig. 6A and B). Bioinformatic analysis using the JASPAR database identified two putative iron-responsive elements (IREs) within the 3′ untranslated region (UTR) of *FOXF1* mRNA, with the consensus sequence CAGWGH (W = A or U; H = A, C, or U) [14]. The mechanism of FOXF1 mRNA regulation by iron is likely analogous to that of divalent metal transporter 1 *(DMT1*) and *Transferrin Receptor 1 (TFR1)*, where elevated intracellular iron promotes binding of iron to iron regulatory proteins (IRPs), triggering their dissociation from the IREs in the mRNA 3′ UTR and leading to decreased mRNA stability [7].

Fig. 6C and D showed that BLM treatment induced increases in both LIP and ROS levels in human primary lung fibroblasts. This observation aligns with previous reports demonstrating that BLM can bind iron and oxygen to form an activated complex capable of releasing cytotoxic oxidants in proximity to DNA polynucleotide chains [15]. Under these conditions, supplementation with FAC further augmented intracellular iron and ROS levels. Importantly, BLM treatment reduced FOXF1 protein levels, while FAC supplementation further suppressed its expression (Fig. 6E). Notably, treatment of BLM-exposed cells with N-acetylcysteine (NAC), which reduced ROS levels without significantly altering intracellular iron (Fig. 6F and G), restored FOXF1 expression (Fig. 6H). Collectively, these data demonstrate that FOXF1 expression is coordinately suppressed by elevated iron and ROS levels during the progression of PF.

### 3.5 FOXF1 decreases ROS and LIP in BLM-treated human primary lung fibroblasts

**Fig. 7:**
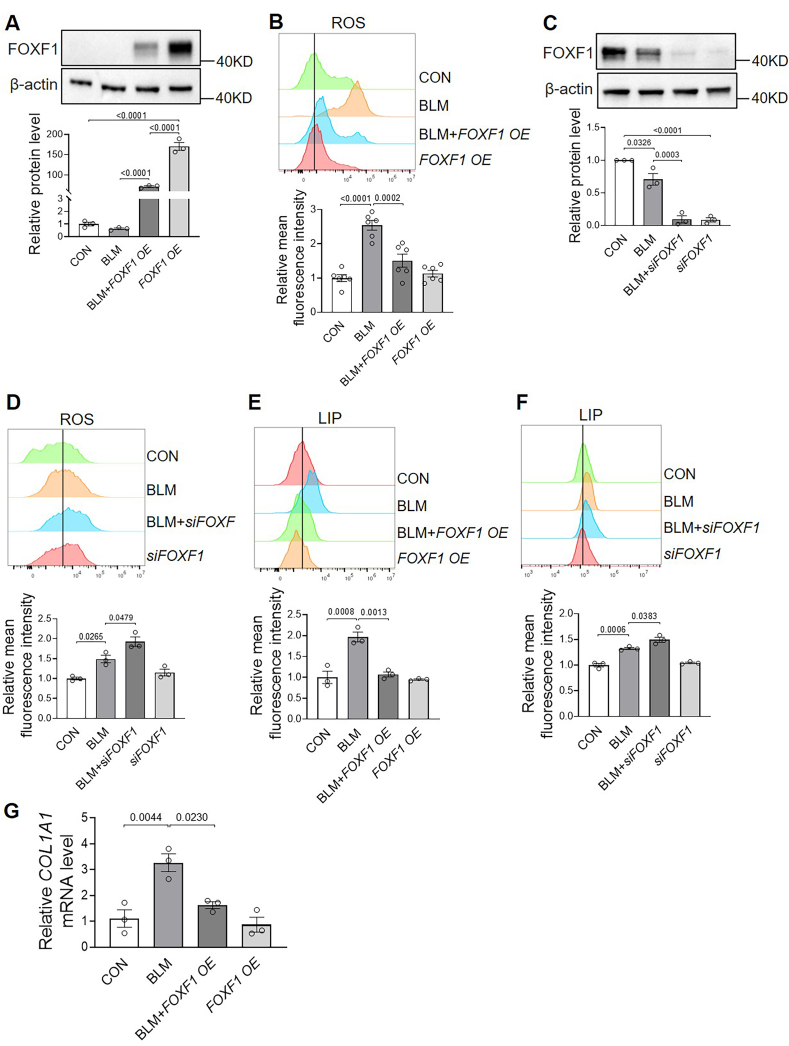
**FOXF1 reduces ROS, iron levels, and *COL1A1* transcripts in BLM-treated human lung fibroblasts.** Cells were treated for 24 h with BLM combined with either *FOXF1* overexpression or *FOXF1* silencing (by *siFOXF1-1*), and ROS and LIP levels were analyzed by flow cytometry using DCFH-DA and FeRhoNox-1, respectively. A, FOXF1 protein expression and quantification following *FOXF1* overexpression. B, Intracellular ROS levels measured by flow cytometry. C, FOXF1 protein expression and quantification following *FOXF1* silencing. D, ROS levels following *FOXF1* silencing. E, LIP levels after *FOXF1* overexpression. F, LIP levels after *FOXF1* silencing. G, *COL1A1* mRNA levels analyzed by real-time PCR using *β-actin* as an internal control. BLM, 40 μg/mL. n ≥ 3/group.

To investigate whether FOXF1 regulates oxidative stress and intracellular iron levels in human lung fibroblasts, we measured ROS and LIP levels following the overexpression or silencing of *FOXF1*. As shown in Fig. 7A–F, FOXF1 modulation did not affect basal levels of ROS or LIP in human primary lung fibroblasts. However, upon BLM treatment, FOXF1 overexpression significantly suppressed the BLM-induced increase in intracellular ROS (Fig. 7A and B), whereas FOXF1 silencing further exacerbated ROS accumulation (Fig. 7C and D). Similarly, FOXF1 overexpression markedly reduced BLM-induced iron accumulation (Fig. 7E), while FOXF1 silencing further elevated LIP levels (Fig. 7F). Additionally, FOXF1 overexpression inhibited the BLM-induced upregulation of collagen mRNA expression in these cells (Fig. 7G).

**Fig. 8:**
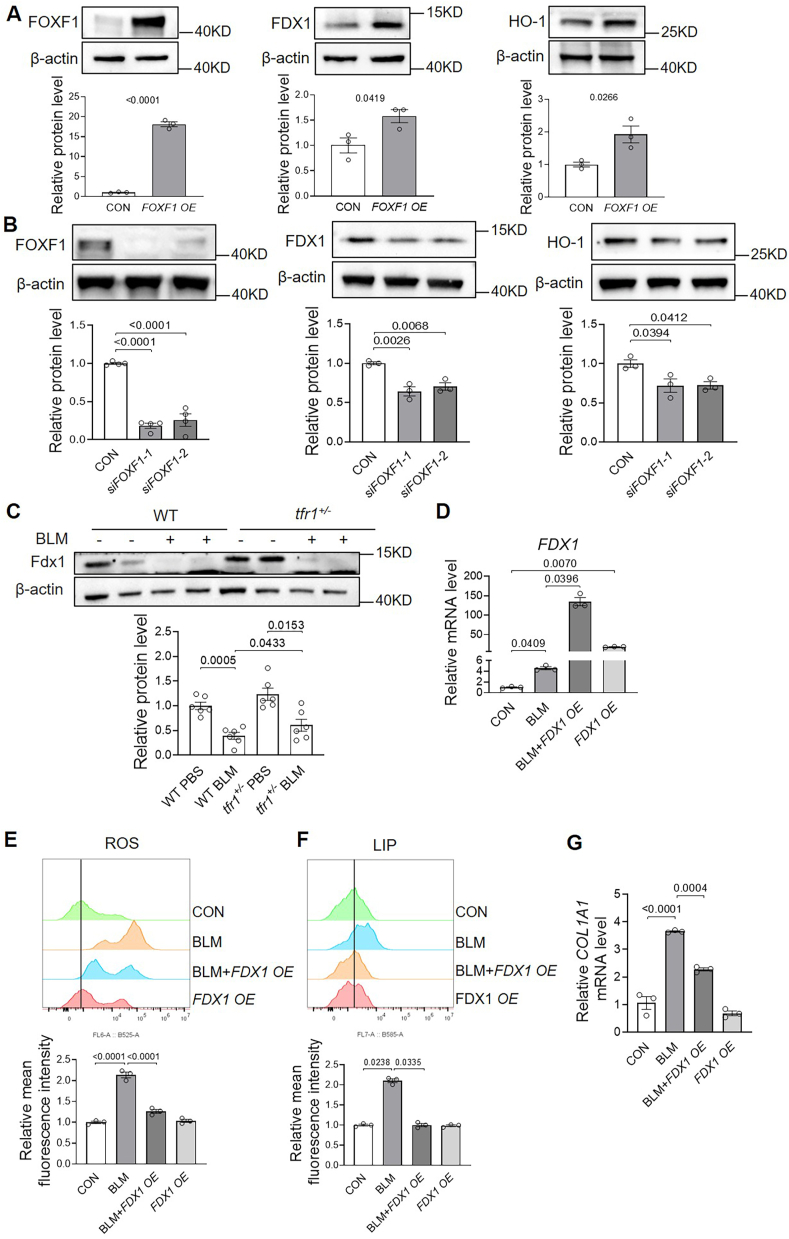
**FOXF1 promotes antioxidant protein expression, and FDX1 decreases ROS, Fe^2+^ levels, and *COL1A1* in BLM-treated human primary lung fibroblasts**. A, The protein levels of FDX1 and HO-1 were analyzed by Western blot in *FOXF1*-overexpressing fibroblasts. n = 3/group. B, Protein expression following *FOXF1* silencing. n = 3–4/group. C, Western blot analysis of FDX1 protein levels in lungs from BLM-treated WT and *tfr1*^*+/−*^ mice with *t*-test. n = 6/group. D–G, Human fibroblasts treated for 24 h with BLM and *FDX1* overexpression were analyzed for *FDX1* mRNA levels by RT-PCR with β-actin as the internal control (D), ROS levels by DCFH-DA probe and flow cytometry (E), LIP levels by FeRhoNox-1 and flow cytometry (F), and *COL1A1* mRNA levels by real-time PCR with β-actin as the internal control (G). BLM, 40 μg/mL n = 3/group.

Previous studies have demonstrated that ROS promote collagen synthesis in fibrotic conditions [16,17]. Therefore, the reduction in ROS mediated by FOXF1 likely contributes to its inhibitory effect on collagen expression. To elucidate the mechanisms underlying FOXF1-mediated ROS suppression, we examined whether FOXF1 regulates the expression of antioxidant-related proteins, particularly FDX1 and HO-1. FDX1, primarily localized to the mitochondrial matrix [18], contains an iron–sulfur (Fe–S) cluster and transfers electrons from NADPH to cytochrome P450 enzymes via ferredoxin reductase (FDXR) [19]. Loss or dysfunction of FDX1 enhances electron leakage from the electron transport chain, thereby increasing ROS production [20]. HO-1 has been shown to attenuate PF and oxidative stress in BLM-induced mouse models [21,22]. As shown in Fig. 8A and B, FOXF1 overexpression significantly upregulated the protein levels of both FDX1 and HO-1, whereas FOXF1 silencing suppressed their expression. These regulatory effects were also observed at the mRNA level (Fig. S4A and B), suggesting that FOXF1 modulates their expression through transcriptional regulation.

Using JASPAR analysis, we identified a FOXF1-binding motif ("RTAAAYA") [23] located 2180 bp upstream of the FDX1 translation start site. Given the concordant changes in FDX1 expression following FOXF1 modulation, our findings strongly suggest that FDX1 is a direct transcriptional target of FOXF1. Supporting this hypothesis, Fdx1 expression was significantly reduced in the lungs of BLM-treated mice compared to PBS-treated controls. In contrast, Fdx1 expression was significantly elevated in *tfr1*^*+/−*^ mice under BLM challenge compared with that of WT (Fig. 8C), a pattern that mirrors FOXF1 expression. Moreover, overexpression of FDX1 in BLM-treated human primary lung fibroblasts reduced both intracellular ROS and LIP levels (Fig. 8D–F) and suppressed collagen expression (Fig. 8G). Collectively, these findings suggest that FDX1 functions downstream of FOXF1 to mitigate oxidative stress, alleviate iron overload, and inhibit collagen synthesis in the context of PF.

### 3.6 SHH elevates iron levels and promotes proliferation and cell cycle progression in human pulmonary fibroblasts

**Fig. 9:**
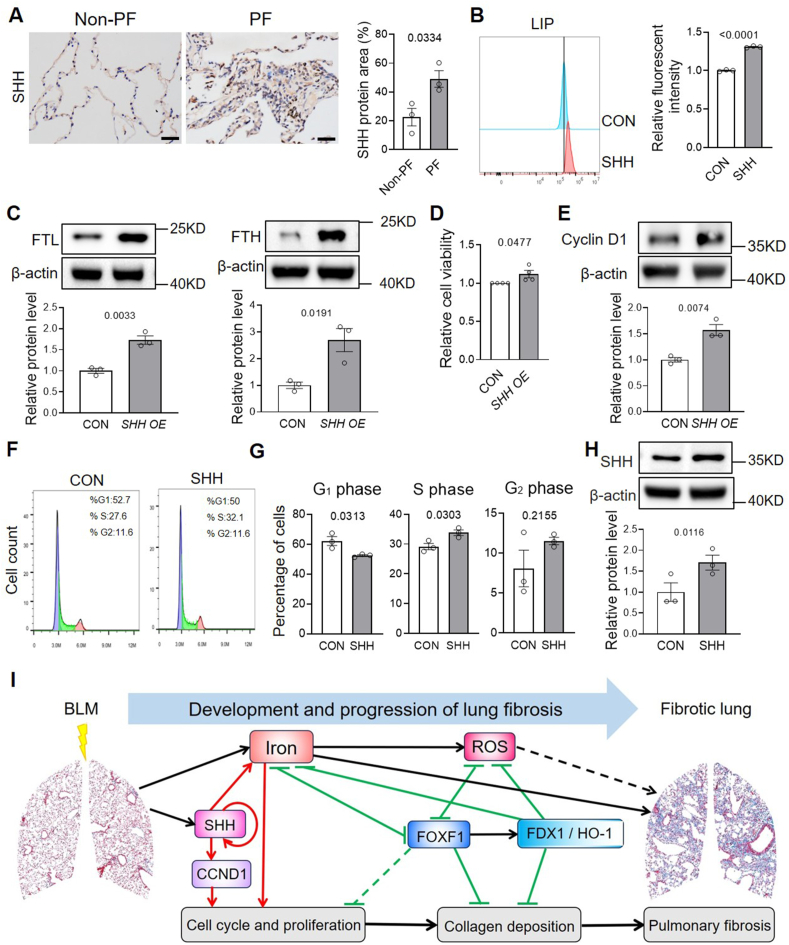
**SHH elevated iron levels and promotes cell viability and cell cycle progression in human primary lung fibroblasts**. A, Representative immunohistochemistry images with anti-SHH and quantification of SHH area/nuclear area by ImageJ in non-PF and PF lung sections. Scale bars, 50 μm. B, LIP levels in fibroblasts treated with exogenous SHH for 24 h were measured using FeRhoNox-1. C, The protein expression and quantification of FTL and FTH were assessed in *SHH*-overexpressing fibroblasts. D, Cell viability results assayed using an MTT Cell Proliferation and Cytotoxicity Assay kit in *SHH*-overexpressing fibroblasts. E, Cyclin D1 protein levels were evaluated by immunoblotting in *SHH*-overexpressing fibroblasts. F and G, Representative cell cycle (F) and quantitative phase distribution (G) were analyzed following 24 h SHH treatment. H, SHH protein levels and quantification were determined after 24 h of SHH treatment at a dose of 250 ng/mL n = 3/group. I, A schematic image demonstrating FOXF1 and SHH in regulating the fibrotic functions of fibroblasts during the development and progression of PF. The solid lines and dashed lines represent the conclusions demonstrated in this study and those previously reported, respectively.

Previous studies have reported that SHH expression is upregulated in fibrotic lungs during PF [24]. SHH promotes the expression of *FOXF1* via GLI transcription factors and activates downstream target genes such as *CCND1* and *CCND2*, thereby facilitating cell cycle progression [25]. Based on this, we hypothesized that SHH expression may interact with intracellular iron levels. To investigate this hypothesis, we first confirmed that SHH expression was significantly elevated in the lungs of patients with PF (Fig. 9A). Treatment with FAC and ferrous sulfate (FeSO_4_) did not significantly alter SHH expression in human pulmonary fibroblasts (Fig. S5A). Furthermore, neither exogenous SHH treatment nor SHH overexpression significantly affected *FOXF1* expression in the cells (Fig. S5B and C). Notably, treatment with recombinant SHH increased intracellular iron levels in human pulmonary fibroblasts by approximately 31 % (Fig. 9B), accompanied by a significant upregulation of FTL and FTH protein levels (Fig. 9C). These findings suggest that SHH signaling promotes intracellular iron accumulation.

In parallel, SHH overexpression promoted the viability of human pulmonary fibroblasts (Fig. 9D). Exogenous SHH treatment significantly increased the proportion of cells in the S phase and decreased the proportion in the G_1_ phase (Fig. 9E and F), indicating enhanced G_1_/S cell cycle progression. This effect of 10.13039/501100017040SHH was further supported by elevated levels of CCND1 in SHH-overexpressing cells (Fig. 9G), and was similar to the effect observed with iron supplementation. Consistently, in murine primary lung fibroblasts, treatment with exogenous SHH at concentrations ranging from 250 to 500 ng/mL accelerated cell viability (Fig. S6A) and upregulated the mRNA expression of pro-proliferative genes such as *HAS2* [26] and *Hmga2* [27] (Fig. S6B).

Interestingly, exogenous SHH treatment also significantly increased the expression of endogenous SHH in human pulmonary fibroblasts (Fig. 9H), suggesting that SHH promotes its own expression through autocrine or paracrine mechanisms. Therefore, SHH drives fibroblast proliferation, an effect that may be at least partially mediated by its ability to elevate intracellular iron levels.

A schematic overview of the findings in this study is presented in Fig. 9I.

## 4 Discussion

Disruption of pulmonary iron homeostasis has been previously reported in patients with IPF, characterized by excessive iron accumulation in lung tissue [28]. In the present study, we observed elevated expression of FTL and FTH in human fibrotic lung tissues. Pulmonary iron levels in BLM-PF mice increased from day 7 post-BLM instillation until harvest, which was in line with fibroblast activation and fibrogenesis during PF development. Notably, by day 21 post-BLM administration, compared to control mice, significantly elevated iron levels were observed in four primary lung cell types: epithelial cells, endothelial cells, macrophages, and mesenchymal cells, indicating a widespread cellular increase in iron accumulation within fibrotic lungs.

Previous studies have shown that alveolar macrophages from *Dmt1*-deficient mice exhibit increased iron loading and worsened BLM-induced lung injury [29]. Similarly, mice with *Tfr2* mutations or *Hfe* deficiency exhibit enhanced pulmonary iron deposition, more severe tracheal fibrosis, and impaired lung function [9]. *Tfr1*^*+/−*^ mice demonstrated reduced organ iron content [30]. In the current study, iron-supplemented BLM-treated mice exhibited higher lung iron levels, increased collagen deposition, and elevated α-SMA expression. Conversely, BLM-PF mice treated with an iron chelator or possessing *Tfr1* haploinsufficiency showed markedly decreased iron levels and reduced collagen accumulation and α-SMA levels. These findings support a pro-fibrotic role for iron in lung remodeling, which is in agreement with earlier studies. Increased numbers of mesenchymal cells (Pdgfα^+^) were also observed in BLM-PF murine lungs, and iron enrichment and depletion increased and decreased mesenchymal cell numbers, respectively. Together with the iron-dependent regulation of α-SMA, these findings suggest that iron facilitates both fibroblast proliferation and fibrogenic differentiation. In vitro experiments further confirmed that elevated iron promotes fibroblast proliferation and accelerates G_1_/S cell cycle progression, whereas iron deprivation inhibits these processes.

We also demonstrated that BLM exposure increases both LIP and ROS levels in human lung fibroblasts. To elucidate the underlying signaling pathways, we focused on FOXF1, a member of the Forkhead Box transcription factor family. FOXF1 downregulation has been shown to upregulate genes associated with cell cycle progression, including *CCND1, CCNB1, CDK1, PEA15*, and proliferation-related gene *ATX* [11]. Notably, the relationship between FOXF1 and iron levels had not been previously explored. Our results demonstrate that both iron overload and oxidative stress suppress the expression of FOXF1. Conversely, FOXF1 overexpression reduces ROS and LIP levels, while FOXF1 knockdown exacerbates them in BLM-treated human lung fibroblasts. These findings suggest a positive feedback loop in which FOXF1 downregulation perpetuates elevated ROS and iron levels. Along with that, ROS mediates TGF-β1–induced fibroblast activation and collagen synthesis [31], and FOXF1 knockdown promotes cell cycle–related gene expression [11]. It is strongly suggested that the positive loop among FOXF1 suppression, iron, and ROS elevation sustains fibroblast proliferation and collagen production in PF.

Iron overload also produced hydroxyl radicals via the Fenton reaction. ROS-induced oxidative stress damages mitochondria, further amplifying ROS production and promoting intracellular iron influx [32,33]. In our study, iron supplementation suppressed FOXF1 expression and increased ROS in BLM-treated human lung fibroblasts, and antioxidant treatment with NAC upregulated FOXF1 and decreased LIP levels with a trend, suggesting a FOXF1-mediated mechanism by which iron and ROS amplify each other in fibrotic conditions.

In addition to promoting ROS generation, loss of FDX1 activity disrupted the activity of iron-sulfur cluster enzymes and cellular iron homeostasis, causing mitochondrial iron overload [34]. It is in consensus that FDX1 overexpression attenuated both ROS and LIP levels in BLM-treated fibroblasts in this study. FDX1 has not been previously reported to regulate collagen levels. Our findings reveal that its overexpression significantly suppresses collagen type I alpha 1 chain (*COL1A1)* expression, likely through ROS reduction. Furthermore, FOXF1 was shown to regulate *FDX1* expression at both mRNA and protein levels, along with the existence of FOXF1 binding motifs within the *FDX1* promoter region, suggesting that *FDX1* is a downstream effector of FOXF1 in controlling LIP, redox status, and fibrogenesis. FOXF1 also promotes the expression of another antioxidant gene, *HO-1*, thereby reinforcing its role in reducing oxidative stress and promoting collagen synthesis. Importantly, FDX1 has been reported to inhibit cancer cell proliferation [35,36], suggesting it may contribute to the acceleration of fibroblast proliferation in FOXF1-downregulated fibroblasts. Together, these findings highlight the FOXF1–FDX1 axis as a critical regulator of oxidative homeostasis and fibrotic remodeling.

SHH is the only Hedgehog family member expressed and secreted in the lung, where it regulates organogenesis and development [37]. SHH signaling is markedly upregulated during PF progression [38] and is produced by epithelial cells, macrophages, and resident mesenchymal cells [37,39,40]. Upon binding to the Patched (PTCH) receptor of neighboring cells, SHH relieves the inhibition of Smoothened (SMO), stabilizing GLI transcription factors and promoting their accumulation in the nucleus, thereby activating target cell cycle-related genes, including *CCND1, CCND2,* and *CCNE1,* which further promote cell cycle progression [25]. Consistent with this pathway, our study showed that SHH enhances fibroblast proliferation, accelerates G_1_/S transition, and upregulates CCND1, which mimics the effects of iron. We found that SHH increases intracellular iron levels in human pulmonary fibroblasts, upregulates the expression of FTL and FTH, and promotes its own secretion. These findings suggest that elevated SHH levels in the lung may regulate iron homeostasis and initiate a self-amplifying loop, which jointly and persistently promotes fibroblast activation, proliferation, and cell cycle progression in both the iron-overloaded lungs of IPF patients and the BLM-induced PF model. Furthermore, iron may mediate the pro-proliferative and cell cycle–acceleration effects of SHH on fibroblasts, a mechanism that could be broadly applicable to various cell types.

Although FOXF1 has been identified as a downstream transcriptional target of GLI proteins, its expression depends on the paracrine SHH signal [25,41]. Neither exogenous SHH treatment nor SHH overexpression altered FOXF1 expression in human lung fibroblasts in our study. We suppose that SHH-induced iron accumulation may antagonize its transcriptional activation of FOXF1. This dual regulatory effect may reflect complex interactions dependent on cell type, experimental conditions, or signaling mechanisms.

## 5 Conclusions

Iron accumulation is a hallmark of PF and promotes fibroblast proliferation, collagen deposition, and disease progression. This study identifies FOXF1 as a key transcriptional regulator that mediates the persistent imbalance of iron and redox homeostasis and fibrogenic differentiation by the iron/ROS–FOXF1–iron/ROS positive feedback loop in pulmonary fibroblasts in PF, at least partly through FDX1. SHH–iron amplification pathway sustains elevated iron level and persistent fibroblast cell cycle progression and proliferation during PF progression. Together, this study delineates a novel molecular regulatory network that links iron metabolism, oxidative stress, and fibrogenesis in PF, offering new targets and avenues for therapeutic intervention.

## CRediT authorship contribution statement

**Xue Wang:** Investigation, Formal analysis, Conceptualization. **Xin Liu:** Data curation, Formal analysis. **Yumei Fan:** Writing – review & editing, Writing – original draft, Conceptualization. **Ke Tan:** Writing – review & editing, Writing – original draft, Conceptualization. **Jiaqi Gao:** Writing – review & editing, Writing – original draft. **Yuejiao Wang:** Investigation, Formal analysis. **Ziyi Zhang:** Investigation, Formal analysis. **Shuyue Liu:** Investigation, Formal analysis. **Xiaofan Wang:** Investigation, Formal analysis. **Baohua Wang:** Writing – review & editing, Writing – original draft, Conceptualization. **Pengxiu Cao:** Writing – review & editing, Writing – original draft, Funding acquisition, Conceptualization.

## Declaration of generative AI and AI-assisted technologies in the writing process

During the preparation of this work, the authors used ChatGPT in order to correct grammatical errors. After using this tool, the authors reviewed and edited the content as needed and take full responsibility for the content of the publication.

## Funding

This work was supported by the 10.13039/501100001809National Natural Science Foundation of China (31900817 and 32071119).

## Declaration of competing interest

The authors declare that they have no known competing financial interests or personal relationships that could have appeared to influence the work reported in this paper.

## Data Availability

Data will be made available on request.
