# Supplementary material for: FOXF1 and SHH participate in the regulation of iron signaling in pulmonary fibrosis
Source: Redox Biol. 2025 Oct 10;87:103893. doi: 10.1016/j.redox.2025.103893 (PMC12553025; doi:10.1016/j.redox.2025.103893)
Supplement: Multimedia component 1 [file mmc1.pdf]

Fig. S1

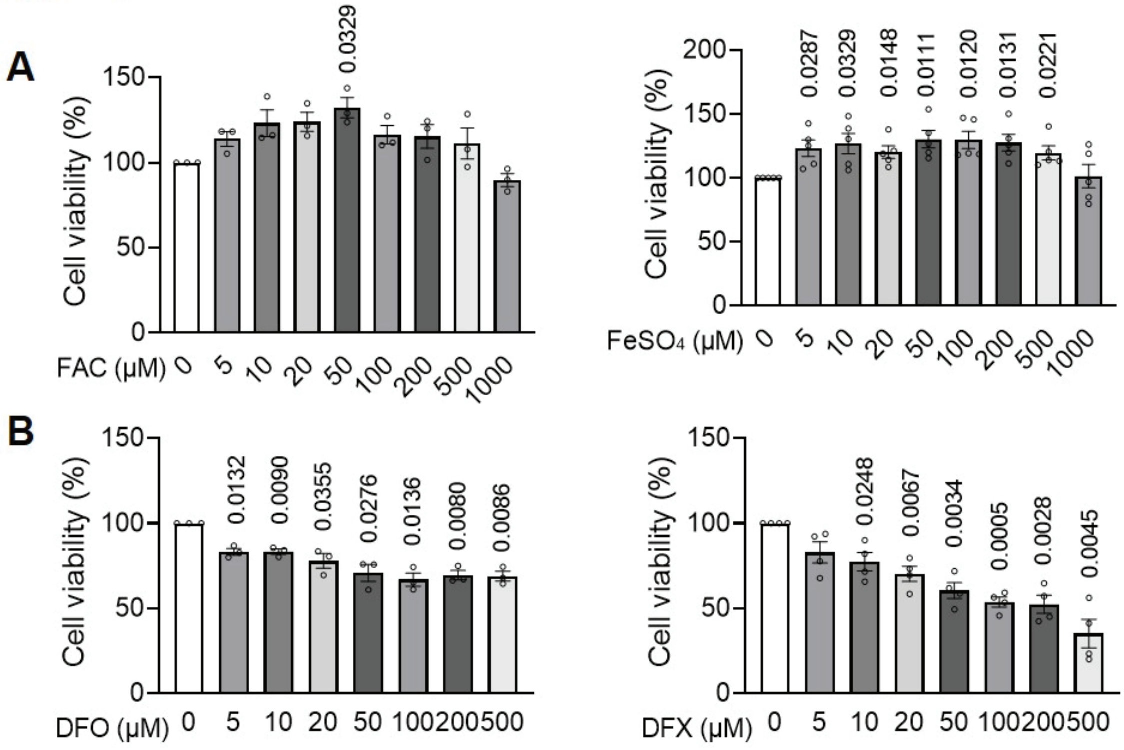

Fig. S1. Iron supplementation enhances cell viability in mouse lung fibroblasts, whereas iron chelation suppresses it. A and B, Primary lung fibroblasts isolated from BLM-PF mice were treated with iron supplements (A) or chelators (B) for 48 h, and cell viability was measured using an MTT Cell Proliferation and Cytotoxicity Assay kit. n ≥ 3/group. T-tests were performed.

Fig. S2

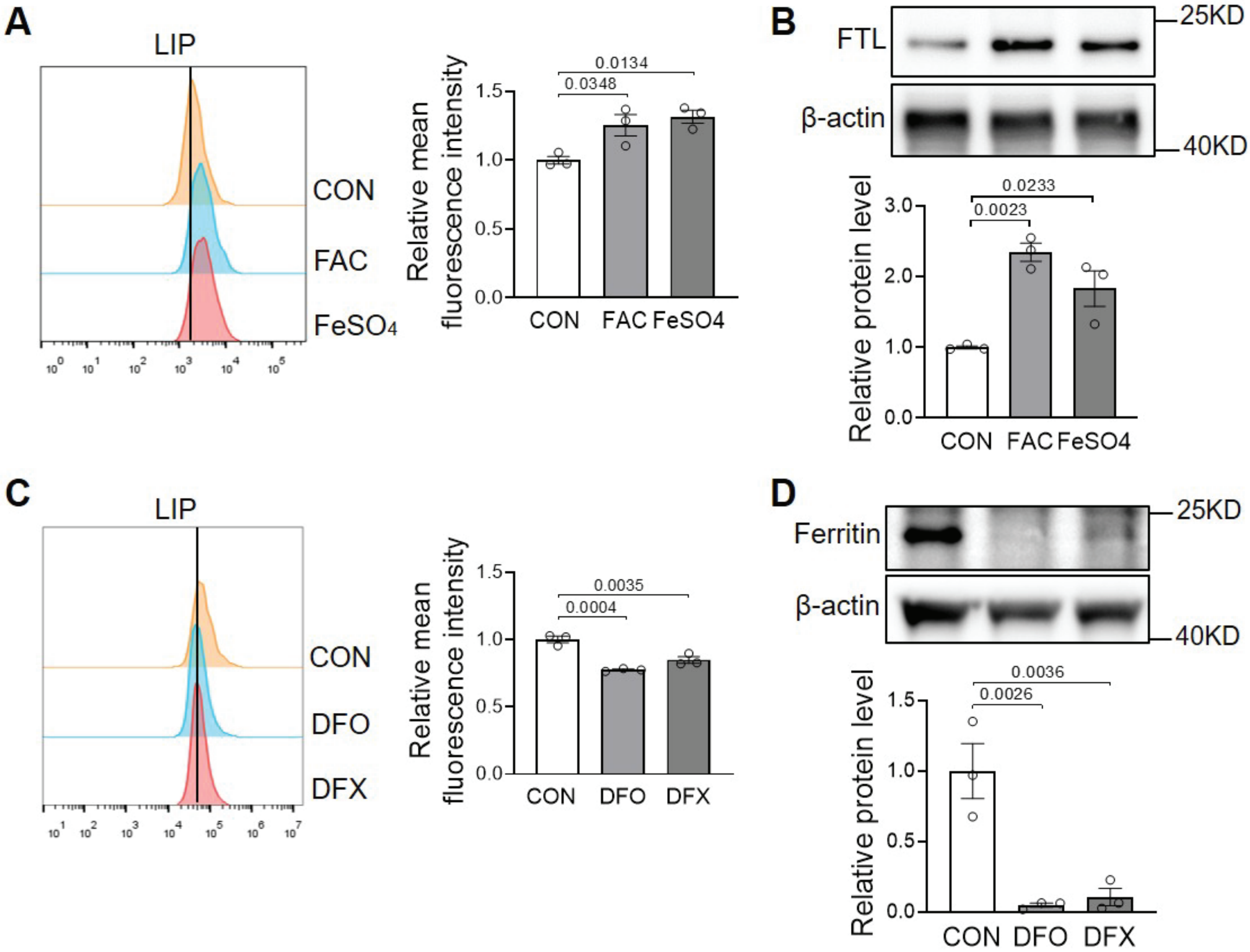

Fig. S2. Iron supplementation increases, whereas iron chelation decreases, intracellular iron levels in human lung fibroblasts. Cells were treated for 24 h, stained with FeRhoNox-1, and analyzed by flow cytometry to determine LIP levels. Also, ferritin protein levels were assessed by Western blotting. A, LIP levels following iron supplementation. B, FTL protein expression and quantification after iron supplementation. C, LIP levels following iron chelator treatment. D, Ferritin protein expression and quantification after iron chelator treatment. FAC (200 μM), $FeSO_4$ (100 μM), DFO (200 μM), and DFX (200 μM); n = 3/group.

Fig. S3

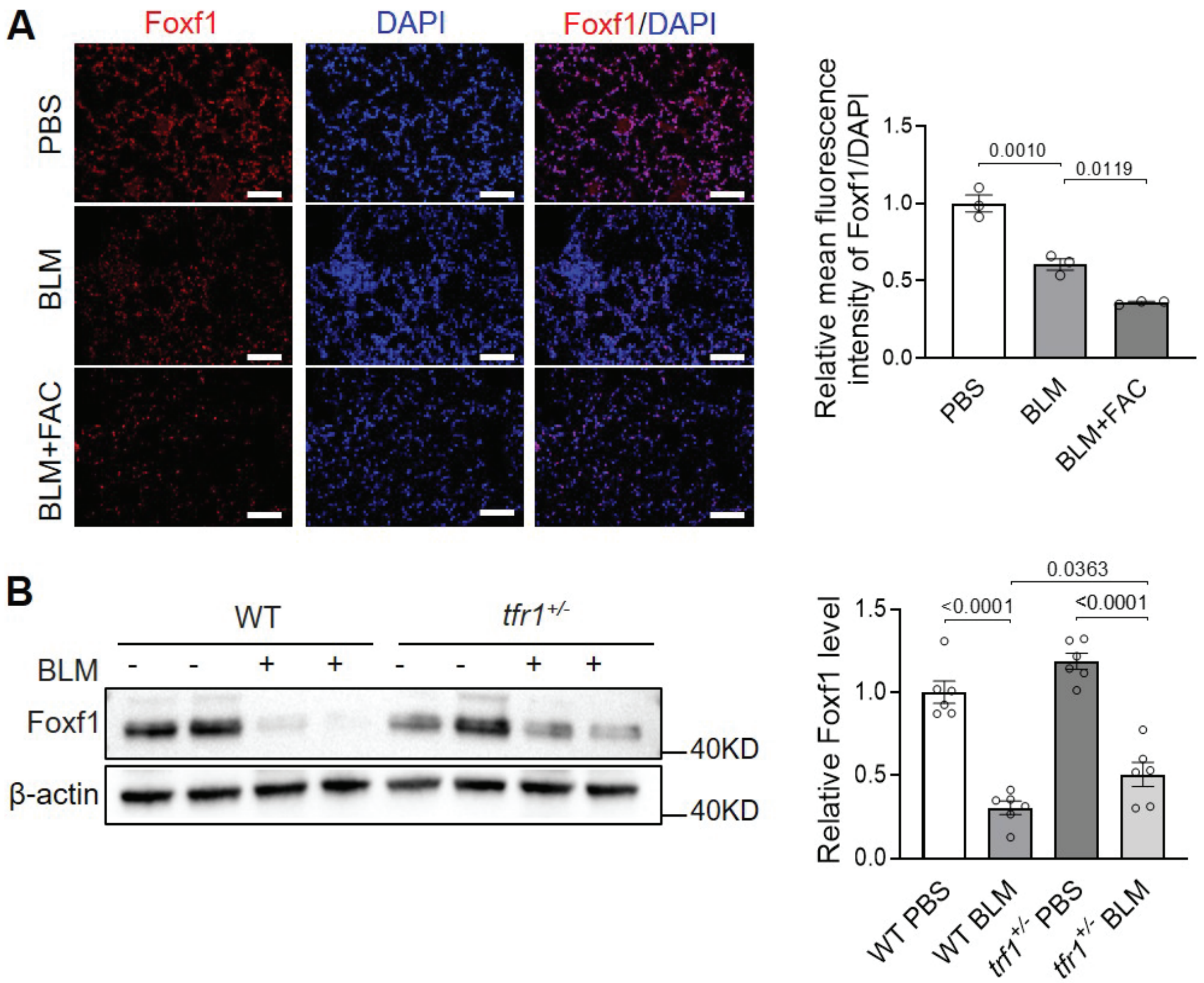

Fig. S3. Elevated iron suppresses FOXF1 expression in BLM-PF mouse lungs. A, Starting from day 7 post-BLM instillation, mice in the BLM+FAC group were administered FAC (600 mg/kg) daily via oral gavage, and lungs were harvested on day 21. Representative FOXF1 immunofluorescence images and quantitative analyses are shown. n = 3/group. Scale bars, 100 μm. B, Western blot analysis of FOXF1 protein expression in lungs from WT and *tfr1*$^{+/-}$ mice at day 21 post-BLM with ImageJ quantification by unpaired t-test. n = 6/group.

Fig. S4

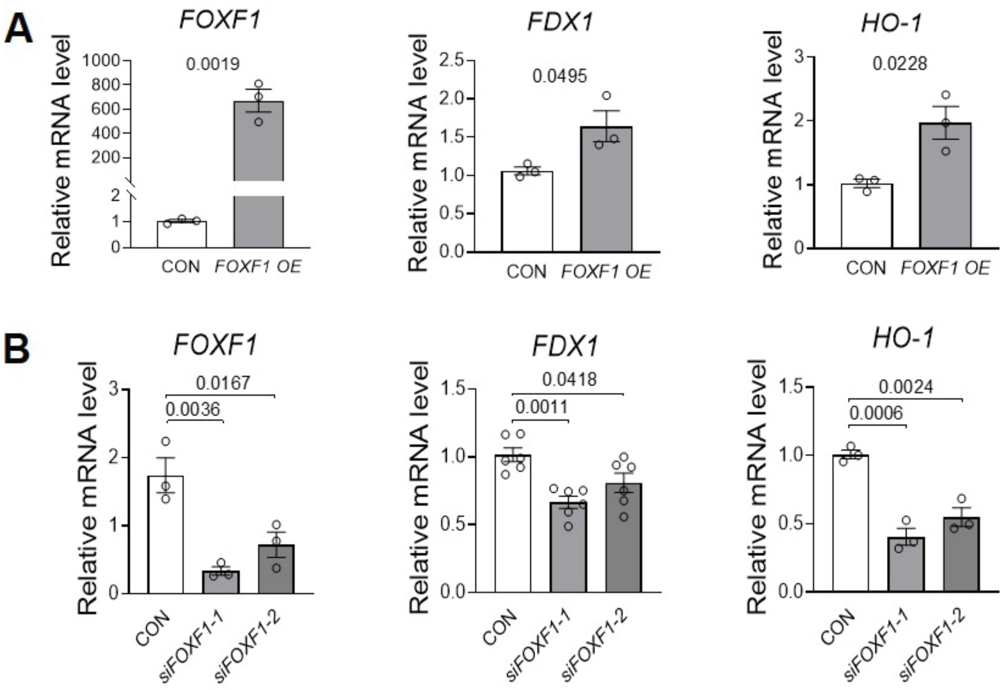

Fig. S4. FOXF1 increases *FDX1* and *HO-1* transcript levels in human lung fibroblasts. A, Cells overexpressing *FOXF1* were analyzed by real-time PCR for the indicated mRNAs using *18S rRNA* as an internal control. B, *FOXF1* silencing cells were analyzed for the indicated transcript levels by real-time PCR using β-actin as an internal control. n ≥ 3/group.

Fig. S5

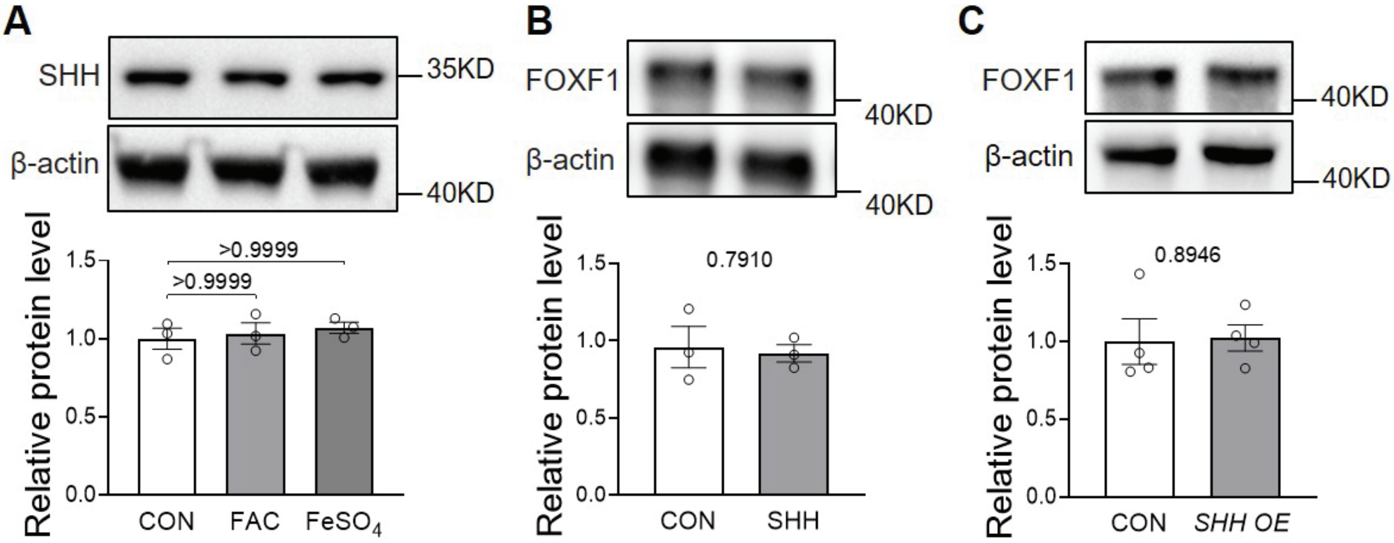

Fig. S5. Iron does not alter SHH levels, and SHH does not affect FOXF1 levels in human lung fibroblasts. A, Cells treated with FAC (200 μM) or $FeSO_4$ (100 μM) for 24 h were analyzed for SHH protein expression by Western blot. B and C, Cells treated with exogenous SHH (B) or *SHH* overexpression (C) for 24 h were analyzed for FOXF1 protein expression. n ≥ 3/group.

Fig. S6

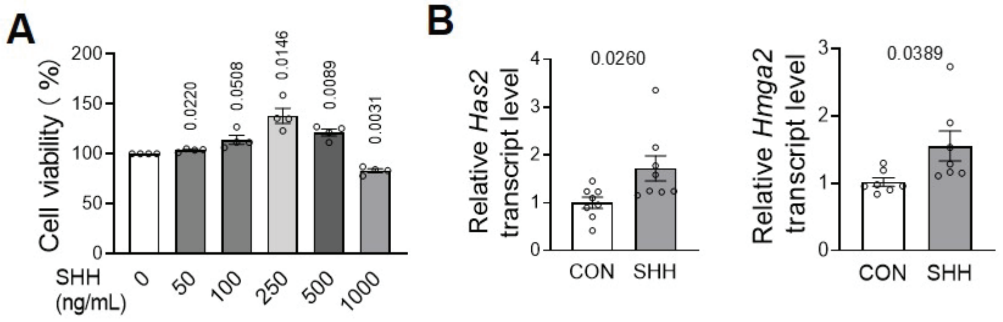

Fig. S6. Exogenous SHH protein promotes the viability of mouse lung fibroblasts. A, Cell viability was measured by an MTT Cell Proliferation and Cytotoxicity Assay kit after exogenous SHH treatment for 48 h with t-test. n = 4/group. B, Proliferation-related gene mRNA levels were assessed by real-time PCR after SHH treatment for 6 h using *18S rRNA* as an internal control. n = 7-8/group. SHH: 250 ng/mL.
